# Mobile Apps for Weight Management: A Review of the Latest Evidence to Inform Practice

**DOI:** 10.3389/fendo.2020.00412

**Published:** 2020-06-24

**Authors:** Drishti P. Ghelani, Lisa J. Moran, Cameron Johnson, Aya Mousa, Negar Naderpoor

**Affiliations:** ^1^Monash Centre for Health Research and Implementation, School of Public Health and Preventive Medicine, Monash University, Melbourne, VIC, Australia; ^2^Diabetes and Vascular Medicine Unit, Monash Health, Melbourne, VIC, Australia

**Keywords:** mobile applications, obesity, mHealth, weight management, weight

## Abstract

Over the last decade, mobile technology has emerged as a potentially useful platform to facilitate weight management and tackle the current obesity epidemic. Clinicians are being more frequently asked to give advice about the usefulness of mobile apps and many individuals have already integrated apps into their attempts to manage weight. Hence, it is imperative for clinicians involved in weight management to be aware of the latest developments and knowledge about available mobile apps and their usefulness in this field. A number of newly published studies have demonstrated promising results of mobile-based interventions for weight management across different populations, but the extent of their effectiveness remains widely debated. This narrative literature review synthesizes the latest evidence, primarily from randomized controlled trials (RCTs), regarding the clinical use of mobile applications for weight management, as well as highlight key limitations associated with their use and directions for future research and practice. Overall, evidence suggests that mobile applications may be useful as low-intensity approaches or adjuncts to conventional weight management strategies. However, there is insufficient evidence to support their use as stand-alone intensive approaches to weight management. Further research is needed to clarify the extent of utility of these applications, as well as the measures required to maximize their potential both as stand-alone approaches and adjuncts to more intensive programs.

## Introduction

With increasing knowledge of the health risks of obesity, appropriate weight management in the present day has become more critical than ever before. Overweight and obesity have been steadily increasing across the global population over the last 50 years, and the need for intervention has become increasingly pressing due to the high burden of comorbidities associated with increased body weight, and the significant challenges of sustained lifestyle modification (1). In 2016, ~39% (1.9 billion people) of the adult population was overweight, including 13% (650 million) being obese (2). This population are at a high risk of many associated comorbidities including type 2 diabetes, cardiovascular disease, fatty liver disease, obstructive sleep apnoea, musculoskeletal disorders (such as osteoarthritis) as well as certain types of cancer (2) leading to reduced quality of life (3). This increases both the complexity and the immediate need for effective weight management. Despite an ever-increasing abundance of weight management programs and products attempting to reduce obesity, substantial, and long-term weight loss has not been achieved, with the exception of pharmacotherapy including glucagon-like peptide-1 (GLP-1) analogs (4, 5) or more drastic measures such as surgery among the extremely obese in general or those with associated comorbidities such as diabetes (6). Paradoxically, obesity rates continue to rise despite a large proportion of the population dieting at any given time.

With such a bleak prospect, it is both timely and necessary for the integration of new technologies to revolutionize healthcare and healthy living, both by practitioners and the public. The emergence of new technology and digital accessibility to health information has brought more awareness of the importance of lifestyle modification in addressing the current obesity epidemic. Although technological advancements such as phones, tablets, and computers have become part of everyday life, the scope, and potential of these now commonplace technologies are yet undefined in the context of weight management, or in healthcare more broadly. Electronic health (eHealth), defined by the World Health Organization as the use of information and communication technologies for health (7), is responsible for the digitalization of medical records and has made the transmission of information among healthcare practitioners and between health practitioners and patients easier and more cost-effective (8). Mobile Health (mHealth), a subset of eHealth, refers to the use of mobile wireless technologies for health (9). mHealth applications have varying degrees of specificity to their target audiences, with many of them aiming to aid patients with complex diseases, and others to simply help healthy adults maintain a balanced lifestyle (10). Indeed, the considerable increase in the number and availability of these mHealth products on the market means that patients and consumers are becoming increasingly aware of healthy lifestyle choices and are able to reflect and monitor their own health behaviors on a regular basis (11).

The rapid emergence of eHealth and mHealth technologies is paralleled with new evidence both supporting and discrediting the use of these approaches for weight management. Hence, there remains a lack of clarity regarding the potential for these technologies to transform the treatment of obesity and lifestyle modification and whether they are able to meet the needs of both the patient and the healthcare practitioner. Moreover, the large number of applications available, coupled with the demanding nature of healthcare services, means that practitioners often do not have the time to evaluate the merits and shortcomings of mHealth applications. An evaluation of the existing evidence with respect to the use of mobile applications for weight management is therefore timely and highly relevant to be able to ensure their effective use within healthcare settings.

The purpose of this narrative literature review is to synthesize the latest high-level evidence, primarily from randomized controlled trials (RCTs), to shed some light on the progress of the field to date and inform the use of such applications, limitations, and challenges associated with their use, as well as recommendations for future research and practice. This review is not systematic and is not intended to present new data or conclusions, but instead aims to summarize what is currently known on this topic in a form which is compact and accessible to practitioners in this field.

## Qualitative Assessment of Factors Influencing the Use of Mobile Applications for Weight Management

**Figure 1:**
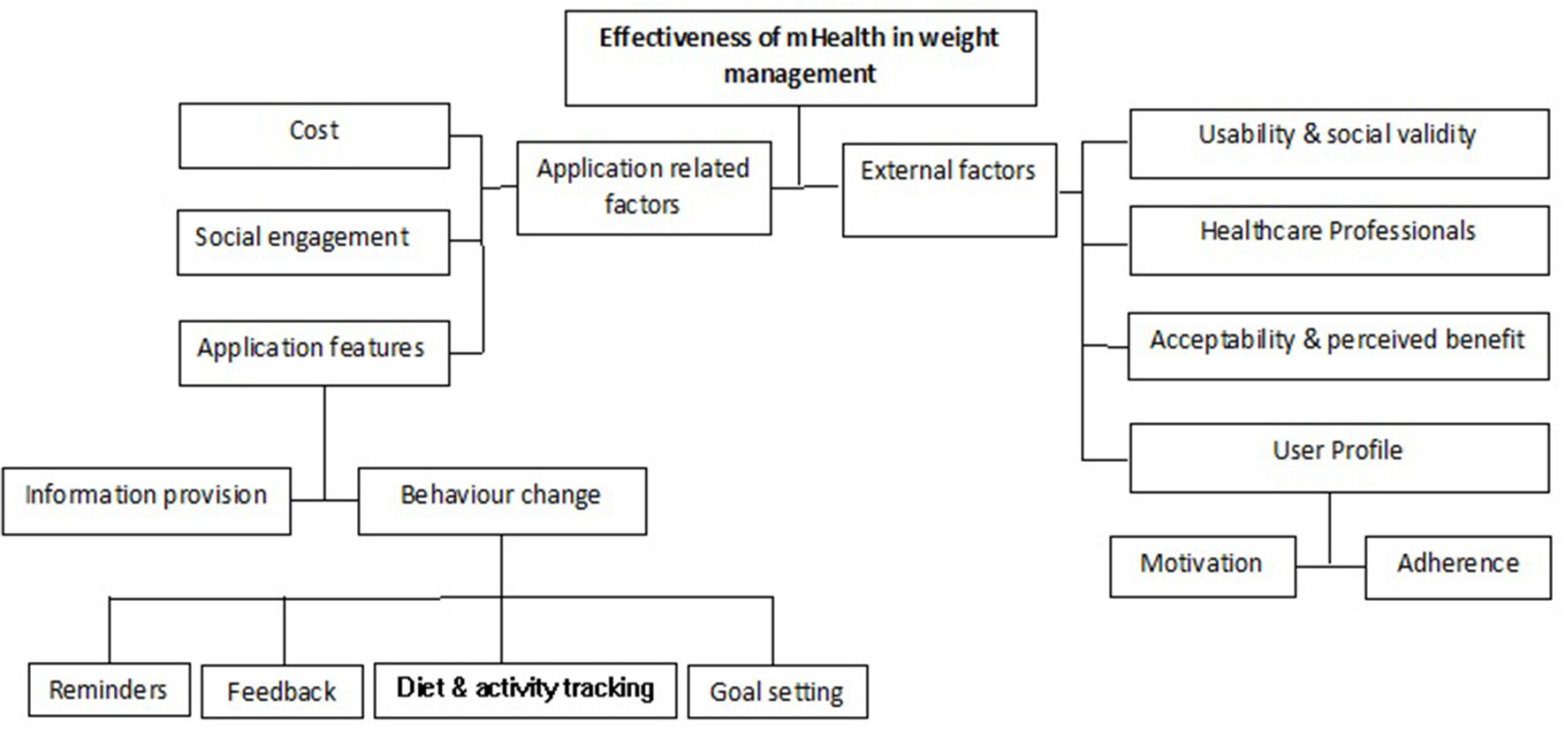
Factors influencing the effectiveness of mHealth in weight management.

To date, there have been many studies examining different aspects of applications for weight management and from the standpoint of both users and healthcare providers. Many of these are reviews which look into stakeholder perspectives and experiences, rather than collecting quantitative data. While they represent a small proportion of aspects to consider when using and designing weight management applications, the information extracted from these studies is nonetheless important. Our review of the existing literature has highlighted the key aspects of mHealth applications into broad categories as described briefly below and illustrated in Figure 1.

### Acceptability and Perceived Benefit

Mobile applications for weight management often have similar features that include self-monitoring of diet and physical activity, allowing users to set goals in specified time frames, feedback on daily activities, and reminders to input data into the application in the form of in-app reminders or text messages. To gauge the general opinion of mHealth application users, a study interviewed adults who had used smartphone applications to examine the perceived effectiveness of such an aid in managing diet (12). When asked for feedback on their use of commercially available weight management applications, participants indicated that they perceived their eating habits, and weight management to be better following application use. The interviews outlined heterogeneity in the applications and users. Where some users entered meal data after eating, others had pre-planned diets, and others had end-of-day logs at home despite having portable devices. Some participants found the entry process much faster with a mobile application (compared to paper records or websites), yet others found it time consuming and discontinued its use. Additionally, some participants used the applications when they noticed poor habits and discontinued their use when they felt they were back on track, while others had more consistent use. Self-motivation was found to be an important factor for effective application use and behavior change, particularly given that the use of these applications required effort and organization, and that adherence was often difficult to maintain (13). Other factors including achieving positive results, identifying streaks in data recording, and even paying for the app were shown to motivate individuals to continue using them.

Some studies have specifically measured long-term adherence to mobile applications in comparison to paper- or website-based diaries. For example, a pilot study aimed to test the acceptability and feasibility in terms of recruitment, dropout, and adherence of a mobile application called “My Meal Mate.” This study showed that retention declined over 6 months and that attrition was unequal between the control web-based diary and the mobile application groups. The most likely reason was speculated to be the type of intervention, with participants noting that disliking the type of intervention was the most cited reason for not attending follow ups (14). Adherence to application use itself was tested in another RCT that assessed within-person daily and seasonal influences on self-monitoring behaviors over 6 months (15). The results suggested that participants recorded fewer items, fewer calories, and less fat in their diet but overall lower diet quality on weekends. Participants also reported fewer food items as the study progressed which may be related to either fatigue with the intervention demands, or success with weight loss from eating fewer foods and thus having fewer items to record. Physical activity measured using a pedometer was lower on weekends than weekdays.

In the perspectives explored, there was an apparent expression of interest from patients asking healthcare professionals for recommendations on applications to use (11). In the current era of precision medicine and targeted therapy, considerations need to be made when developing and using these applications to increase efficiency and reduce risk of unpleasant or non-significant outcomes. It should also be noted that in many of these existing studies, the applications being tested are those developed by researchers that are not available to the public (16). This is slowly changing, where applications like eaTracker® (which was developed for research in collaboration with dietitians) are now making it onto the Canadian market (17). Many of the study designs have also been criticized, where samples are too small and not representative of the general population, as well as questioning whether the use of applications by participants in a research environment or context is translatable to a real-life setting (18, 19).

### Information Provision

The nature of information provided by mHealth applications covers a range of topics required to lead a balanced lifestyle. These include advice on dietary intake, importance of different food groups, physical exercise, energy expenditure, recipes, consequences of not having a healthy life, and everyday tips and tricks to manage weight. Many applications go a further step and include instructions for meditation or mindful living (20, 21). Furthermore, some applications can provide the users with information on their body mass index (BMI) or body fat percentage and help them track changes (22, 23). However, with mHealth applications being relatively uncharted territory, there have also been concerns regarding data accuracy and privacy in these applications. When being developed, many of these applications have little to no input from experts on diet or physical activity (24). This then can lead to inaccurate and unreliable information (25, 26). In addition, many of these applications bypass the physical examinations conducted by the doctors, making the legal implications of such a means highly questionable. There are also legal implications of these gaps in content accuracy and information dissemination that need to be considered. While there are currently regulations from governing authorities in place for mHealth applications, there does not seem to be enough enforcement (27).

### Behavior Change

In a study evaluating the features of weight management applications, the formula for a successful weight-loss application to lead to behavioral change was broken down into twenty features that the application must incorporate. These features include: weight-loss goal, dietary goal, calorie balance, physical activity goal, exercise safety, benefits of healthy diet and physical activity, food pyramid, stimulus control, portion control, lifestyle activity, target heartrate, problem solving, stress reduction, relapse prevention, negative thinking, social cues, developing a regular pattern of eating, time management, and nutritional label reading (28). On average, only 18.8% of these 20 strategies were reflected among 30 applications for weight loss, with the highest percentage seen in MyNetDiary and MyNetDiary Pro (65%), followed by two other applications, All-in Fitness and Noom Weight Loss (both 25%).

In a focus group conducted by Solbrig et al. (29), it was reported that people trying to self-manage their weight using an application had problems with staying motivated due to a lack of time or energy, slow results or getting bored, and not being able to resist cravings. Users reported self-monitoring to be useful at the beginning of commencing lifestyle change, and then sapping rather than strengthening motivation when sustained. Some feared negative attention and therefore did not report their diet or activity on the application. While reporting of dietary intake was correlated with increased likelihood of achieving behavior change and reaching lifestyle goals, reinforcement, and motivation rather than a continued supply of information have been found to be the most desirable qualities of the applications by users trying to lose weight (29). Authors concluded that staying motivated was the most difficult aspect for individuals trying to self-manage their weight and that there was a mismatch between the help provided by weight loss campaigns (information, self-monitoring) and the help needed by individuals (autonomous and motivational e-support) (29). Simply providing health information at regular intervals was shown to be ineffective and the weight that was lost with the intervention was often gained back over the course of a few years (30).

### Usability and Social Validity

Another key strategy that has been highlighted is the social validity of the application in question which revolves around the user's perception and response to the application (31). Levels of satisfaction and engagement are key measurement parameters. High engagement levels of the application correlate to higher levels of adherence as well as resultant weight loss from the intervention. The effectiveness of a given application is therefore seen as equally important to its social validity and the overall user experience. This is supported by a study examining different application features in the weight-loss outcomes of overweight and obese adults (32). A “supportive” application included providing information, monitoring consumption, rewards, prompts and reminders, and personal compliance review with the program, while a “static” application only provided recipes and weight loss information. When the applications were independently used along with personal support, the “supportive” application had lower attrition rates, but no difference in weight loss between the groups.

### Social Engagement

A largely successful feature seen in some popular applications such as BodySpace is the inclusion of a platform resembling social media within the application (33, 34). These applications, together with the normal features including goal setting and monitoring diet and activity, are based upon a platform where application users can “follow” other users who were in similar situations as themselves, reducing feelings of isolation that people often experience at the beginning of a weight-loss journey. There is also an “inspirational” feature where users can follow others on the application whom they found motivational. The social media aspect also allowed users to share their progress as well as other content within the community.

## Empirical Evidence from Randomized Controlled Trials on Mobile Apps for Weight Management

As summarized in Table 1, few RCTs have examined the effectiveness of weight management applications, which makes the current status and trajectory of the field unclear for important stakeholders. In the following sections, we collate the evidence from RCTs and meta-analyses of RCTs that measure various endpoints such as BMI, body weight, and physical exercise to measure the effectiveness of mobile applications for weight management in overweight or obese adults (45, 46). The RCTs examined show a high heterogeneity in the interventions used for control groups. While some studies used modified versions of applications, others measured applications against predetermined standard programs. Yet others examined the use of the applications in addition to existing programs. Unpacking these RCTs and meta-analyses allows us to identify the areas where mHealth applications have attempted to replace or augment conventional means of weight management, and whether they were effective in doing so.

**Table 1:** Characteristics and outcomes of randomized clinical trials examining the usefulness of mobile apps in weight management.

**References**	**Participants**	***n***	**Intervention** **(+used App)**	**Strategy used** **for change**	**Study duration**	**Objective**	**Effect on anthropometry**	**Other outcomes**
Brindal et al. (32)	Overweight and obese adults	146	*I*_1_ = Static app: recipes and weight loss info; *I*_2_ = Supportive app: *I*_1_ + food intake records, rewards, reviews, reminders	Education, self-monitoring, motivation	24 weeks	Observe effects on weight loss, weight-related biomarkers, and psychological outcomes	No differences in weight loss; ~60% of all participants lost ≥5% of body weight	Reduction in app usage lower in supportive app users; ~39.0% of users were still using the app at week 24.
Patrick et al. (35)	Overweight adults	75	*I* = C + brief monthly phone calls, personalized SMS/MMS messages 2–5 times daily; *C* = monthly weight control printed materials	Education, reinforcement for improved behavior	4 months	Observe effects on weight	Greater weight loss in intervention group after adjusting for sex and age [−1.97 kg difference; average weight loss = 2.88 kg (3.16%) with intervention]	NA
Orsama et al. (36)	Patients with type 2 diabetes aged 30–70 years	48	**Monica application**	Self-monitoring + feedback	10 months	Develop and evaluate mobile phone-based remote patient reporting and automated feedback system to improve self-management and health	Greater weight reduction with intervention (−2.1 vs. −0.4 kg)	Greater mean reduction in HbA1c of −0.40%
			*I* = Remote patient health reporting + linked health behavior change feedback; *C* = Standard care (diabetes education and counseling)					
Oh et al. (37)	Patients with metabolic syndrome	405	**Smart Care service**	Self-monitoring, minimizing time and space restrictions	24 weeks	Assess weight loss and adherence effects	Improved body weight, BMI, body fat percentage, waist circumference among active participants compared with less active, or control participants	No difference in lipid profile changes
			*I* = App used to transmit daily information on body composition and physical activity; *C* = No app used					
Allen et al. (38)	Obese adults	68	***Lose it!*** **application**	Self-management, mindful empowerment, real time feedback, and motivators	6 months	Evaluate feasibility, acceptability, and efficacy of behavioral interventions delivered by smartphone technology	Weight loss and reduced BMI was greater in *I*_2_ (−5.4 kg; −1.8 kg/m^2^) and *I*_3_ (−3.3 kg; −1.1 kg/m^2^) compared with counseling alone (*I*_1_ = −2.5 kg; −0.8 kg/m^2^) or app alone (*I*_4_ = −1.8 kg; −0.7 kg/m^2^)	NA
			*I*_1_ = Intensive counseling intervention; *I*_2_ = Intensive counseling plus *Lose it!*; *I*_3_ = A less intensive counseling plus *Lose it!*; *I*_4_ = *Lose it!* Only					
Nollen et al. (39)	Low-income, racial/ethnic-minority girls aged 9–14 years	51	*I* = App based on behavioral weight control principles; *C* = No intervention	Self-monitoring, real time goal setting, providing feedback, and reinforcement	12 weeks	Pilot study examining the effect of a mobile technology as a stand-alone intervention to prevent obesity	No change in BMI	Trends toward increased consumption of fruit and vegetables (+0.88, *p* = 0.08) and decreased sugar sweetened beverages (−0.33, *p* = 0.09). No change in screen time
Burke et al. (40)	Overweight and obese adults	210	**Dietmate Pro**	Self-monitoring	24 months	Examine the effect of PDA on weight loss and maintenance	No differences in weight loss between groups, PDA + feedback group lost weight compared to baseline	Adherence to dietary self-monitoring was the strongest predictor of weight loss
			*I*_1_ = PDA; *I*_2_ = PDA + daily feedback; *C* = Paper diary					
Ross and Wing (41)	Overweight and obese adults	80	*I*_1_ = Technology-based tools (TECH); *I*_2_ = Technology-based tools + phone-based intervention (TECH + PHONE); *C* = Standard self-monitoring tools	Self-monitoring	6 months	Examine the efficacy of self-monitoring technology, with, and without phone-based intervention	Weight loss differed at 6 months between groups; trend for TECH + PHONE to lose more weight than control (−6.4 vs. −1.3 kg); fewer controls achieved ≥5% loss (15 vs. 44% in the other groups)	Adherence to self-monitoring calorie intake was higher in TECH + PHONE than TECH or controls
Carter et al. (14)	Overweight adults	128	**My Meal Mate application**	Goal setting, self-monitoring, feedback	6 months	Compare acceptability and feasibility of self-monitoring weight management intervention delivered by an app, website, or paper diary	Mean change in weight (*I*_1_ = −4.6, *I*_2_ = −1.3, *C* = −2.9 kg); BMI (*I*_1_ = −1.6, *I*_2_ = −0.5, *C* = −1.0 kg/m^2^); and body fat (*I*_1_ = −1.3%, *I*_2_ = −0.5%, *C* = −0.9%) greater in app group at 6 months	Retention was higher in app group (93 vs. 55% web and 53% diary). Self-monitoring declined over time in all groups
			*I*_1_ = Mobile app; *I*_2_ = Website; *C* = Paper diary					
Pellegrini et al. (15) and Spring et al. (42)	Obese adults aged 18–60 years	96	**ENGAGED application**	Self-monitoring, using a food database of over 50,000 foods and social network features	6 months	Examine effect on weight loss + within-person variation in dietary self-monitoring in the tech-supported group	Greater weight loss in tech-supported and standard than self-guided groups (−5.7 kg vs. −2.7 kg). More participants in the standard group achieved weight loss ≥5% compared to tech-supported group	No difference in weight loss at 12 months. Less recording over time in the tech-supported group. Fewer foods reported on weekends and more foods self-monitored in January vs. October but no seasonal effect observed
			*I*_1_ = Tech-supported (eight group sessions + coaching calls + app); *I*_2_ = Standard (eight group sessions + coaching calls + paper diary); *C* = Self-guided (lifestyle DVDs + paper diary)					
Zhou et al. (43)	Healthy adults	64	*I* = Mobile app with adaptive personalized daily step goals; *C* = Active control with steady step goal of 10,000	Adaptive goal setting, self-monitoring	10 weeks	Evaluate efficacy of an automated phone-based goal-setting intervention using machine learning with no in-person contact or counseling	Weight and BMI were measured but effects after intervention were not reported	Mean daily step count decreased by 390 steps in intervention group vs. 1,350 steps in controls from run-in to 10 weeks. Net difference = 960 in daily steps
Thomas et al. (44)	Overweight and obese adults	276	*I*_1_ = GROUP-based: weekly, biweekly, monthly meetings (6 months), paper diaries, feedback; *I*_2_ = SMART-based: online lessons, feedback, monthly weigh-ins; *C* = CONTROL: paper diaries, feedback, monthly weigh-ins	Self-monitoring	18 months	Assess differences in weight loss between smartphone-based vs. intensive group-based behavioral obesity treatments vs. control	No difference in mean weight change	8-month retention was significantly higher in both GROUP (83%) and SMART (81%) compared with CONTROL (66%)

Conventional methods of weight loss include diet alone, diet and exercise, exercise alone, meal replacements, very-low-energy diets, weight-loss medications, and advice alone. These methods have been shown to improve weight loss outcomes in some obese and overweight populations (47). If an mHealth intervention could essentially have the same components as a face to face intervention, the benefit it offers is reduced contact time, which is potentially more cost-effective. Studies have reported that the use of mHealth applications is effective in many contexts and across several populations. For example, Patrick et al. (35) reported that the use of a text message-based intervention in overweight adults resulted in a reduction in weight (mean change = −1.97 kg) over the course of 16 weeks. This is a modest reduction in weight, and a clinical diet/exercise weight loss study over this time would be expected to result in a clinically relevant 5% weight loss (48). Nevertheless, this indicates that using mHealth applications may be effective as a lower intensity and lower cost approach to weight management. Larger changes will likely be seen with more intensive or in-person delivery formats or a combination of these. Indeed, a pilot RCT by Ross and Wing (41) showed that self-monitoring technologies (Fitbit activity monitor, scale and app) combined with brief phone-based interventions resulted in greater adherence and weight loss compared with standard self-monitoring tools alone (reference book, pedometer, paper-based booklet, body weight scale). While using conventional tools led to 15% of patients having at least 5% weight loss, having mobile technology and telephone support tripled this proportion, resulting in 44% of patients achieving weight loss of 5% or more (41).

Another study conducted by Schiel et al. (49) assessed physical activity and eating habits in overweight or obese children or adolescents. The intervention consisted of the integration of a motion sensor onto a mobile device. The 5 week trial resulted in statistically significant improvements in multiple parameters including BMI, physical activity, and stress management (49). Being able to objectively monitor physical activity correlated with an increase in these activities including running, walking, and cycling. In addition, it was a more accurate means of keeping track of exercise as compared to self-reporting.

Smartphone applications are also shown to be effective in longer trials involving both disease and weight management in patients with type 2 diabetes (36). For example, statistically significant mean reductions in glycosylated hemoglobin levels (−0.4%) and body weight (−2.1 kg) were reported after 10 months in patients who used the Monica application, a mobile phone-based reporting and automated feedback system (36). Participants with type 2 diabetes or type 2 diabetes and hypertension monitored their blood pressure, weight, physical activity, and blood glucose values. The automated feedback system in the Monica application observed trends in the patient-reported parameters and sent feedback and improvement suggestions, helping to promote, and sustain positive behavior change (36).

The effectiveness of weight management applications also extends to other chronic conditions (50). A recent systematic review and meta-analysis of studies assessed the impact of using dietary mobile apps on nutritional outcomes in adults with chronic diseases, such as obesity, cardiovascular disease, diabetes, and risk of breast cancer (50). Results showed a positive change among app users in more than 75% of the included studies for at least one of the nutritional outcomes measured, while >50% saw a medium or large effect size in the measured outcomes including weight loss, reduced BMI, and change in energy intake. There was also a clear correlation of dietary monitoring applications with weight loss, to an extent not seen in paper-based monitoring methods, web-based technologies or no self-monitoring at all. More frequent application use was also correlated with better results. The same conclusion was observed when studying application use in overweight and obese children, as well as another trial looking at obesity management in patients with metabolic syndrome (37, 51). However, we note that these reviews did not delineate which specific application characteristics were associated with greater efficacy in these interventions.

Another meta-analysis of 12 studies by Flores Mateo et al. (45) also found that those who used a mobile application showed significant changes in weight (−1.04 kg) and BMI (−0.43 kg/m^2^) compared with control groups. Control groups consisted of primarily traditional interventions (brochures/booklets, diaries, education) or intensive counseling (45). While this is a modest change, the data should be interpreted with a level of caution as there is substantial variability in the included trials which makes interpretation of the results difficult. For example, some RCTs compared mHealth applications against standard care weight-loss programs, while others compared the intervention against no application, or a modified version of the application.

On the other hand, some RCTs have reported smartphone applications to be ineffective for weight management as stand-alone interventions. For example, Allen et al. (38) observed no statistically significant reduction in weight with the use of the Lose it! application in obese patients, with and without behavioral counseling over six months. The use of the application together with varying intensities of behavioral counseling was more effective as compared to having only a smartphone application or counseling *per se*. The application thus showed potential to be used as an adjunct to other weight management interventions. Another study specifically examined the dietary intake aspect of weight management when using a mobile application to set real-time goals, provide tips, self-monitor, and obtain feedback and reinforcement as compared to a booklet providing health tips and goal-setting advice (39). The 12-week trial resulted in no change in diet, including reducing the consumption of sugar-sweetened beverages or increasing consumption of fruits and vegetables. A third study of 210 overweight/obese adults assessed the difference between a personal digital assistant (PDA) in self-monitoring, either with or without daily tailored feedback, compared to a conventional paper diary over 24 months (40). No differences in mean percentage weight loss was found between the three groups; however, across the groups, increased adherence correlated to greater weight-loss. This study, which took place over the course of 24 months, is one of the few to examine the long term effects of using mHealth applications on weight management and suggests that adherence to self-monitoring may be more relevant than the method used to self-monitor. The data presented in this study highlights the limited long-term data available and emphasizes the need for further longitudinal studies examining the effects of mobile applications on weight management and whether these effects are sustained in the long term.

## Barriers to Using Mobile Applications for Weight Management

A common theme in most RCTs on using mobile applications for weight management is the high attrition rate due to participants discontinuing or decreasing use of the application. While monitoring physical activity is shown to be an easier process with the use of inbuilt motion sensors and pedometers, monitoring diet is much more difficult, accounting for a high proportion of the attrition (52). It is posited that the low adherence is largely due to the considerable effort required to self-monitor diet (14). Each item needs to be individually entered into the application, and a greater variety in the diet requires more information to be entered, thus taking up more time (12). When entering nutritional data, consumers prefer to scan a barcode or have suggested food lists (53). In addition, many applications have too little choice of pre-entered meals in their databases, which often do not include ethnic foods. On the other hand, some applications have too much choice and become confusing, making it difficult for users to choose the right meal item that they consumed. Hence, it is argued that compared with manual paper-based records, having an application which enables manual digital entry of consumed foods adds limited value to the field, especially for subsets of the population such as the elderly (54, 55). While having an application makes it easier to calculate nutritional information, there are now greater software advancements, including being able to photograph a meal to replace the manual entry of data (56). However, the accuracy of this latter method remains under debate (57).

The low adherence evident in studies of mobile applications for weight management is a serious limitation, most importantly because of its the potential to introduce bias in the results. It could also reflect dissatisfaction with the intervention. Overall, low adherence in any weight management intervention could contribute to a lack of weight loss (58). Compared to non-adherent groups, individuals who manage to monitor their diets conscientiously on a long-term basis are successfully able to lose weight (59, 60). This highlights a key difference in the two populations: motivation. In all the RCTs, one variable that researchers have not been able to control for is motivation. While in surveys, all participants express their desire to lose weight, not all of them are willing to put in the effort to make it happen (61). An evaluation of incomplete data in diet and weight loss studies showed that individuals who dropped out had a higher starting BMI and reported poorer health compared to those who completed the study, potentially indicating differing levels of motivation (58).

To address these limitations of existing applications, some studies have tested the feasibility of algorithms that alter daily and weekly goals based on targets achieved previously, in an attempt to increase retention (43). One study assessed as the primary endpoint, the relative change in daily steps from run-in to the 10-week follow-up, measured objectively by the participants' iPhones. The machine learning-based algorithms adaptively personalized goals that were attainable but difficult for users, as compared to the control group who got a standardized daily goal of 10,000 steps every day. In doing so, goal achievement was shown to be 15% higher in the intervention group. Being able to achieve more daily step goals likely enhanced participants' motivation, which may have promoted more physical activity in the following days.

However, these findings were disputed in a study assessing weight loss using a gold-standard intensive weight-loss program compared to smartphone-based or control programs over 18 months (44). An initial weight loss goal of 10% of current body weight was set for all groups. The gold-standard method consisted of weekly, biweekly, and monthly visits to dietitians and exercise physiologists for three 6 month blocks. The smartphone intervention had the same information delivered by means of an application, with a variety of short videos being released at designated times and monthly weigh-ins. The standard care group had paper diaries, written feedback, and monthly weigh-ins. Adherence was highest in the gold-standard (83%) and smartphone (81%) groups, compared with controls (66%), but there was no difference in mean weight change between the three groups (5.9, 5.5, and 6.4 kg, respectively), although the former is intensive and is associated with a high monetary cost, making it less feasible outside of a trial setting. It should also be noted that participants in the smartphone and control groups attended monthly weigh-ins, which may have provided additional accountability and motivation compared to a context without regular monitoring.

In interpreting the findings from RCTs, it is important to minimize bias or overgeneralization of results (62). Most of the study designs examined in this review contain implicit bias due to their convenience sampling methods. Trials are advertized using mainstream methods of recruitment such as posters, emails, and internal recruitment from universities or companies, which leads to a reduced capacity for the results to be generalized. With a constant outpouring of new information from studies designed to inform both researchers and consumers, it is vital that high quality data is produced and analyzed, as well as data that has real world applications beyond a trial setting.

Thus, at present, while smartphone applications for weight management are being used by some clinicians including more than half of dietitians in some studies (63), they are not a formal or structured part of therapy. Dietitians mostly use these applications for information and recommend their use to patients for self-monitoring, but the field requires more data and regulation before mHealth interventions can be fully incorporated into therapy. The sheer number of applications and the process of familiarizing themselves with different applications is a daunting process. In addition, many physicians currently using mobile applications to monitor their patients' health often do so free of charge, potentially causing other clinicians to be slightly hesitant in joining them (64). However, when it comes to weight management of overweight or obese patients, physicians are often unable to get involved in the patient's recovery due to the length and complexity of the process. There are several issues contributing to the involvement of health professionals, including lack of confidence, training, time, and stigma, to name a few. As such, referral to other allied health professionals for support in weight management is recommended in evidence-based guidelines for obesity management (for example, in chronic disease management plans or mental health plans) (65). The mantle of seeing the transition to a healthy lifestyle through falls to these allied healthcare professionals, including dietitians, exercise physiologists, and psychologists. In this case, knowledge of appropriate referral pathways for a multidisciplinary approach, and understanding of the roles of allied health professionals who specialize in the area is paramount.

Another potential limitation of mHealth is that many applications with good social validity have an associated financial cost. With the influence of popular culture and icons of these industries, applications like “Centr, by Chris Hemsworth” have taken off with rapid success (66). However, the subscription cost for applications like these is a potential barrier to a large proportion of the population accessing their content (12). This poses a problem in itself due to a perception that applications that cost more are of better quality for their purpose (67).

It is important to acknowledge that providing information about eHealth and mHealth is only one of the steps involved in preparing clinicians and allied health practitioners to accept and integrate technology in their practice. According to a recent framework for assessing and implementing strategies for dietitians' readiness for eHealth, there are five aspects to the readiness to incorporate eHealth into clinical and dietetic settings. These start with having access to the technology, followed by standardization, then the attitude (knowledge about the benefits and appreciating the need and willingness to use technology), aptitude (skills and training to use technology), and advocacy in supporting the initiative (68). In a recent study involving dietitians, after learning of the potential and the benefit of mHealth applications, many were eager to at least test the application to assess its effectiveness (69). The study showed the feasibility and efficacy of a training workshop for dietitians to increase their interest and confidence in integrating mobile applications into their practice. In addition to dietitians, patients included in the study were interested to continue to use and to recommend using the applications for weight management to others. Smartphone applications are perceived favorably due to the low cost associated, accessibility, familiarity, and application-based support whenever there is connectivity (12). Some of these apps including MyFitnessPal® and Calorie Counter have taken off with astonishing success, which opens doors to developing this potential in a much more impactful manner in future (70, 71).

## Future Directions

While the studies conducted thus far have brought the field a long way in understanding the impact of mHealth applications on the ever-increasing burden of obesity, there is still much to be done. Based on the findings of the studies above, the first step required moving forward would be to break down and analyze those traditional weight-loss programs which were successful, in order to uncover the different aspects that applications can attempt to replicate to ensure efficiency. Most trials examined have shown that mobile applications were effective or had great potential to be effective for weight management. However, low user adherence hinders our ability to adequately assess these applications, since the results never seem to reach their full potential. One key aspect that needs to be replicated is evoking the motivation and accountability that is seen in interactions from personal trainers. For example, in the study by Carter et al. (14), a control group participant, when made aware of a weight loss application, downloaded the application and went on to lose 32 kg over the course of the trial, while the mean weight loss of the treatment group was 4.6 kg. The intrinsic motivation of a user who downloads the application needs to be tapped into and maintained. Despite the theoretical and demonstrated efficiency, these applications will not be able to reach their full potential without overcoming the barrier of motivation and adherence.

When put into practice, it is of utmost importance that application features include goals to be achieved, monitoring of physical activity, diet and progress, reminders, feedback, and most importantly, features to ensure continued use. Whether these be positive reinforcement, behavioral counseling, or accountability, depends on what the user needs. There is currently a lack of applications catering toward the social aspect of weight-loss with social support and encouragement from peers on the same journey. Thus, despite the new and constantly improving technology that can bring the practice of weight management forward, the evidence has highlighted once again the multifaceted nature of the weight-loss and management process. The ideal patient who reaps the most benefit from mHealth applications would be one invested in their own weight management, engaged in the application which caters to their specific needs, and has dietary, psychological, and physical support when needed.

Notably, most of the research reviewed took place within developed Western societies, and seems to be applicable across these societies. Most of the popular mobile applications for weight management have been created in developed countries including the USA, Australia, and the UK (25). However, the problem of obesity and the need for weight management is almost worldwide. To date, research into applications has focused on specific subpopulations, limiting the generalizability or global relevance of current findings (31). A need for further, and more widely known applications catering toward the needs of groups with socio-economic restrictions has become apparent. Furthermore, mHealth applications need to be approved and certified by relevant health authorities before they can be integrated into routine medical management within clinical practice. Except for a few countries, there is currently a lack of acknowledgment of the role of mHealth in weight management guidelines and national policies with no established regulations on the use of mobile applications by clinicians (72). Where the use of smartphone technology has been incorporated into national health programs as part of the slow shift toward digital health, they are used more as a means of providing information than a tool to encourage behavior change (73).

In future, mobile applications for weight management should be expanded to other groups of people who could benefit from them, including post-menopausal women and individuals with cancer or diabetes, with research suggesting lifestyle modifications as first-line treatment (74–76). Other populations such as those with anorexia nervosa or the elderly may also benefit from these mobile applications, but the needs of these groups have not been addressed as yet (77, 78). With a rapidly aging and increasingly technologically savvy population, studies conducted have reported the elderly's enthusiasm to occupy their time with socializing and physical activity (79). However, the reach of technology has hitherto escaped this population who could benefit from an intervention to improve physical and mental health and ease the aging process in a vibrant social setting.

## Conclusions

In summary, the use of mHealth interventions for weight management is an emerging field for both research and clinical care. The evidence presented, while mostly positive, remains unclear. Although some studies report that mHealth technology is a step forward in upscaling interventions and bringing them to the public (31), others argue that these applications have limited use and add little value to current intervention options. One of the pressing problems in the use of these applications appears to be in identifying effective ways to promote behavior change and increase motivation in the subset of the population with poor adherence or for whom the applications have not been effective. Arguably, those population groups who are difficult to reach may be at most risk and innovative strategies are thus needed within mobile applications to target these individuals. Behavioral components in particular, including self-monitoring and tailored feedback, are key in any weight management intervention (face to face or mHealth), and optimizing these would make the existing technology go much further in managing weight than any technical improvement. Reducing the effort required to self-monitor would also be required in order to effectively target those populations. While inbuilt mobile technology has increased the ease of regulating and monitoring physical activity, the time-consuming, and relatively difficult input of dietary data onto mobile applications has seen a less than enthusiastic response. In general, mobile applications show potential, albeit more as adjuncts to conventional interventions or low-intensity approaches rather than as intensive stand-alone interventions. There is also good overall satisfaction from consumers, and many believe they have benefited from the use of mHealth applications. The main implication from the current evidence is that while the technology available now is useful, there remains considerable untapped potential in the area. Although most studies converge on the key features of a successful weight management application, there remains a lack of empirical support to demonstrate the effectiveness of these applications across populations and over time.

## Author Contributions

DG synthesized and collated the literature, reviewed the evidence, wrote the first draft, edited, and revised the manuscript. LM and CJ contributed to editing and writing the manuscript. AM contributed to scoping and structuring the review, supervising the process, writing, and editing the manuscript. NN conceptualized the review topic, contributed to scoping and reviewing the evidence, supervising the process, writing, and editing the manuscript. All authors provided substantial intellectual input to the work in line with ICMJE criteria for authorship and approved the final manuscript for publication.

## Conflict of Interest

The authors declare that the research was conducted in the absence of any commercial or financial relationships that could be construed as a potential conflict of interest.
